# Repurposing Dapagliflozin for Mitigation of the Kidney Injury Triggered by Cadmium in Rats: Role of Autophagy, Apoptosis, and the SIRT1/Nrf2/HO-1 Pathway

**DOI:** 10.3390/ph17121690

**Published:** 2024-12-15

**Authors:** Hany H. Arab, Musaad M. Althobaiti, Abdulaziz S. Alharthi, Emad O. Almalki, Saif S. Alsoubie, Jawad M. Qattan, Saeed A. Almalki, Ahmed M. Ashour, Ahmed H. Eid

**Affiliations:** 1 Department of Pharmacology and Toxicology, College of Pharmacy, Taif University, P.O. Box 11099, Taif 21944, Saudi Arabia; 2 College of Pharmacy, Taif University, P.O. Box 11099, Taif 21944, Saudi Arabia; 3 Department of Pharmacology and Toxicology, College of Pharmacy, Umm Al Qura University, P.O. Box 13578, Makkah 21955, Saudi Arabia; 4 Department of Pharmacology, Egyptian Drug Authority (EDA)—Formerly NODCAR, Giza 12654, Egypt

**Keywords:** AMPK, autophagy, cadmium, dapagliflozin, nephrotoxicity, SIRT1

## Abstract

**Background/Objectives:** The antioxidant/antiapoptotic features of dapagliflozin (DPG) have mediated its beneficial actions against several experimental models. However, no studies have been conducted to determine whether DPG mitigates the renal injury triggered by cadmium (Cd). Herein, DPG was studied for its potential to attenuate kidney damage in Cd-intoxicated rats, as well as to unravel the mechanisms involving oxidative events, autophagy, and apoptosis. **Methods:** Histopathological analysis, immunohistochemical staining, and ELISA were conducted on kidney tissue samples. **Results:** Cd administration (5 mg/kg/day; p.o.) prompted significant renal damage, as evidenced by histopathological changes, elevated kidney injury molecule-1 (KIM-1) expression, and increased serum creatinine and urea. Interestingly, DPG (1 mg/kg/day; p.o.) significantly mitigated these harmful effects without affecting renal Cd metal accumulation. Mechanistically, DPG curbed Cd-induced renal pro-oxidant response and stimulated the antioxidant sirtuin 1 (SIRT1)/nuclear factor (erythroid-derived 2)-like 2 (Nrf2)/heme oxygenase 1 (HO-1) axis. Moreover, DPG restored autophagy by decreasing sequestosome-1/protein 62 (SQSTM-1/p62) accumulation and stimulating the AMP-activated protein kinase (AMPK)/mechanistic target of rapamycin (mTOR) pathway. In tandem, DPG suppressed Cd-induced apoptosis by lowering renal Bcl-2 associated-x protein (Bax) and cytochrome C (Cyt C) levels and caspase 3 activity. **Conclusions:** These findings indicate that DPG attenuates Cd-induced nephrotoxicity by enhancing the SIRT1/Nrf2/HO-1 antioxidant pathway, promoting AMPK/mTOR-directed autophagy, and inhibiting apoptotic cell death.

## 1. Introduction

As a toxic heavy metal, cadmium (Cd) is a widespread pollutant derived from anthropogenic activities [1,2]. The main entry portals into the body for cadmium are the digestive and respiratory systems, as well as direct skin contact [1,3]. The kidney is a key target organ for Cd poisoning. Typically, patients with cadmium-induced nephrotoxicity exhibit significant proximal tubular dysfunction characterized by excessive excretion of low-molecular-weight proteins and electrolytes in the urine [2]. In the kidney, cadmium has a mean half-life of 14 years. This long half-life is due to its retention in large amounts in renal tubular cells [3].

Numerous mechanisms have been proposed to explain cadmium-induced renal damage, including increased oxidative stress and apoptosis in renal proximal tubular cells [4,5]. Cd induces cellular oxidative stress by disrupting the balance between the oxidative and antioxidative systems. Consequently, reactive oxygen species (ROS) are excessively generated and deplete the antioxidant defense system of the cell, which further aggravates the oxidative damage [2,3]. Several studies have been conducted in vivo and in vitro demonstrating that Cd increases renal lipid peroxidation, decreases antioxidant enzyme activity, and depletes the nuclear factor (erythroid-derived 2)-like 2 (Nrf2) [6,7,8] and sirtuin 1 (SIRT1) expression in rodent models of Cd-evoked nephrotoxicity and cadmium-triggered renal tubular cytotoxicity in vitro [5]. The depletion of SIRT1 in kidney tissues has been linked to excessive pro-oxidant events and apoptosis [5,9].

In eukaryotic cells, autophagy plays an integral role in the survival, homeostasis, development, and differentiation of cells. This occurs through the degradation of cellular dysfunctional organelles, macromolecules, and proteins, and the recycling of their degradation products [10]. In the same regard, the process of apoptosis involves blistering of the plasma membrane, cell shrinkage, and chromatin condensation/fragmentation [11]. Numerous studies have revealed that autophagy and apoptosis contribute to the development of Cd-induced nephrotoxicity in mammals [7,12,13,14,15]. When exposed to stressors, the cells respond initially by augmenting the autophagy events to protect renal tubular tissues from damage at an early stage. Yet, prolonged exposure to Cd results in lysosomal alkalinization that inhibits autophagy events and cargo degradation, resulting in renal tubular cell apoptosis [11,16]. In renal pathologies, conflicting reports have been published regarding autophagy events. In perspective, different nephrotoxic agents have been shown to stimulate [17] or disrupt [18,19] renal autophagy flux. In terms of cadmium toxicity, conflicting data have been reported about its ability to modulate autophagy flux during in vitro studies of proximal tubular cells [1]. Likewise, experimental models of Cd-induced renal injury revealed overactive [20] as well as impaired autophagy events [8,10] in vivo in animals. Hence, additional research on the in vivo effects of Cd on the renal tissues is still needed.

**Figure 1:**
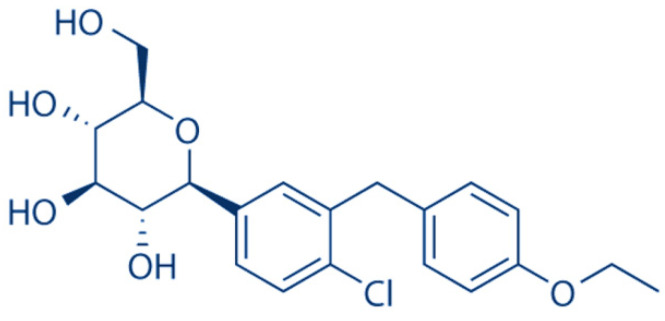
Illustration of dapagliflozin’s chemical structure.

Dapagliflozin (DPG; Figure 1) is a medication prescribed for type 2 diabetes mellitus patients that acts as an inhibitor of the sodium-glucose cotransporter 2 (SGLT2) in the kidney. By limiting glucose reabsorption from renal filtrate, DPG can improve blood glucose fasting and postprandial levels [21,22]. In the control of blood sugar, the risk of hypoglycemia associated with DPG has been reported as minimal [22]. Moreover, several studies have characterized the high safety of DPG, as seen in the toxicology studies reported in rats and beagle dogs [23]. In terms of the tissue expression of SGLT2, the kidney is the primary tissue for its expression [24]. Interestingly, DPG has been reported to exhibit significant antioxidant [25,26,27,28] and anti-apoptotic activities [25,27,28]. These features have advocated its protective potential against several experimental paradigms, such as cardiac ischemia/reperfusion injury [28], inflammatory bowel disease [25], and testicular injury [29]. Moreover, the pro-autophagic actions of DPG and activation of the AMP-activated protein kinase (AMPK)/mechanistic target of rapamycin (mTOR) pathway have been characterized to curb rodent models of lipopolysaccharide-evoked Alzheimer-like manifestations [30], colitis [25], hepatic steatosis [31], and pancreatic injury [32]. In renal pathologies, DPG has been reported to curtail gentamicin-induced renal injury [27], diabetic kidney damage [33], and vancomycin-triggered nephrotoxicity [34]. Moreover, off-label uses of DPG include the management of proteinuric chronic kidney disease and diabetic kidney disorder [35]. However, the possibility of DPG curtailing renal damage instigated by cadmium has not been previously examined. Thus, the present study attempts to bridge this gap by investigating the potential ameliorative prospect of DPG on cadmium-evoked renal damage. Moreover, we explored the molecular events associated with the SIRT1/Nrf2 pathway as well as the AMPK/mTOR cascade.

## 2. Results

### 2.1. Cadmium-Induced Impairment in Renal Function Was Improved by Dapagliflozin in Rats

**Figure 2:**
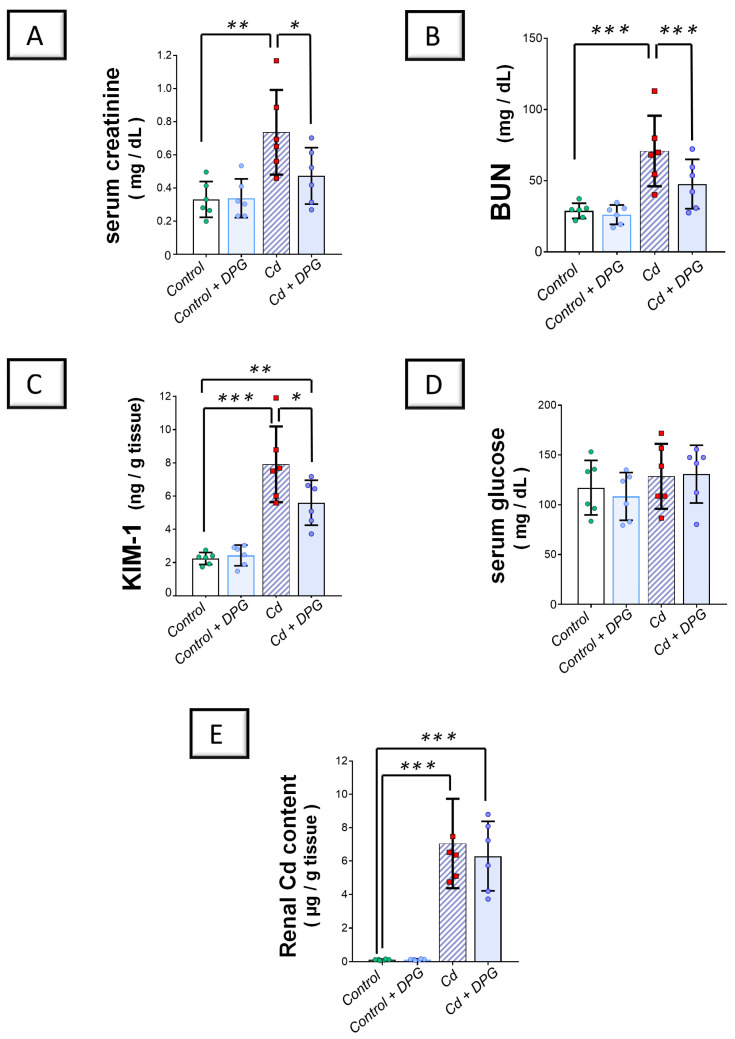
Dapagliflozin improved Cd-triggered kidney dysfunction in rats. These effects were proven by reduced serum creatinine (**A**) and blood urea nitrogen (BUN) (**B**), alongside the dampened renal expression of KIM-1 (**C**), compared to the Cd group. Moreover, serum glucose levels (**D**) and renal Cd metal content (**E**) were examined in all experimental groups (**D**). For *n* = 6, the graph bars represent the mean ± standard deviation. Statistical significance was described by * *p* < 0.05, ** *p* < 0.01, or *** *p* < 0.001. Cd, cadmium chloride; DPG, dapagliflozin.

Investigation of the kidney function markers revealed that Cd impaired the renal function in rats by significantly elevating serum creatinine (*p* < 0.01) and urea (*p* < 0.001) levels by 122.4% and 145.4%, respectively, versus the control group (Figure 2A,B). In the same regard, Cd significantly enhanced the kidney injury molecule-1 (KIM-1) protein expression (*p* < 0.001) by 252.6% in the renal tissues of animals (Figure 2C). According to these findings, the Cd-induced renal injury model was confirmed to be successfully established. Co-administration of DPG with Cd afforded a significant reduction in serum creatinine (*p* < 0.05) and urea (*p* < 0.001) levels by 35.7% and 32.7%, respectively. Likewise, DPG and Cd co-administration elicited a significant lowering in renal KIM-1 expression (*p* < 0.001) by 29.2% in comparison to the Cd group. In addition to the renal function tests, we quantified serum glucose in animals to check whether hypoglycemia may have occurred in normoglycemic rats in response to DPG administration (Figure 2D). Herein, non-significant changes were detected in serum glucose levels in all the groups, denoting that DPG does not elicit hypoglycemia in animals. To examine whether DPG affects the renal excretion of Cd metal content in the kidney tissues, we measured renal Cd content in all experimental groups. Compared to the control group, the renal Cd content increased significantly in the Cd group by 5722% (*p* < 0.001; Figure 2E). However, DPG and Cd co-administration elicited a non-significant change in renal Cd metal content compared to the Cd group, suggesting that dapagliflozin does not promote kidney excretion of Cd. Collectively, these findings advocate that DPG can improve renal function markers associated with Cd intoxication without affecting renal Cd excretion in rats.

### 2.2. Cadmium-Induced Renal Histomorphological Changes Were Relieved by Dapagliflozin

**Figure 3:**
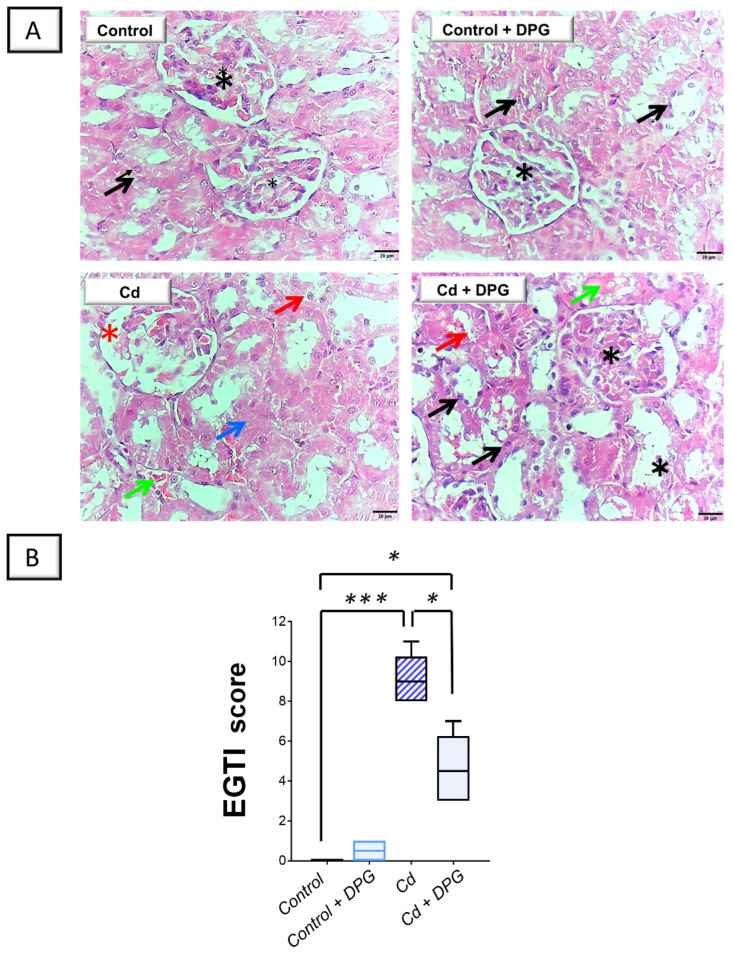
Dapagliflozin improved Cd-induced renal histomorphological aberrations in rats. (**A**) The renal cortex from the control as well as the DPG-treated rats manifested typical histological features, including an intact proximal and distal convoluted tubule epithelium (black arrow) and typical glomerulus (black asterisk; scale bar: 20 µm). On the contrary, the Cd-treated group showed severe atrophy of the glomerular tufts together with an increased Bowman’s space (red asterisk). Moreover, there was evident vacuolation (red arrow), necrosis of the tubular epithelium (blue arrow), and hemorrhage (green arrow; scale bar: 20 µm). DPG and Cd co-administration afforded moderate mitigation of the histological aberrations by improving the size/shape of renal corpuscles and decreasing vacuolization, necrosis, and hemorrhages. (**B**) According to the EGTI’s scoring of renal histomorphological damage, renal tissue demonstrated reduced damage scores in response to DPG co-administration with Cd, implying an improved kidney histopathology. For *n* = 6, the bars represent the median with the interquartile range. Statistical significance was described by * *p* < 0.05 or *** *p* < 0.001. Cd, cadmium chloride; DPG, dapagliflozin.

Staining of the kidney tissue with the hematoxylin and eosin (H-E) stain was applied to dissect the pathological changes prompted by Cd. Figure 3A illustrates the histopathological findings of rat kidneys in all groups. The renal cortex from the control as well as the DPG-treated rats exhibited typical histological features, including an intact proximal and distal convoluted tubule epithelium and typical glomerulus. On the contrary, there was marked morphological damage in the kidney tissue of the Cd-treated group. In perspective, a severe atrophy of glomerular tufts and an increased Bowman’s space were detected. Moreover, there was evident vacuolation, necrosis of the tubular epithelium, and hemorrhage. These findings further reinforce the successful establishment of the Cd-induced renal injury model. As illustrated in Figure 3A, DPG and Cd co-administration afforded moderate mitigation of the histological aberrations by improving the size/shape of renal corpuscles and decreasing vacuolization, necrosis, and hemorrhages in comparison to the Cd group.

As a means of evaluating the severity of histopathological damage, EGTI scoring was utilized to quantify the pathological aberrations (Figure 3B). In this system, four types of histological damage were quantified: endothelial, glomerular, tubular, and interstitial damage. The current findings revealed that renal damage scores significantly increased (*p* < 0.001) following CdCl_2_ administration. When DPG was co-administered together with CdCl_2_, these scores were significantly reduced (*p* < 0.05) by 49.1%, suggesting that DPG is capable of dampening renal histopathological changes.

### 2.3. Cadmium-Induced Renal Pro-Oxidant Events Were Curbed by Dapagliflozin in Rats

**Figure 4:**
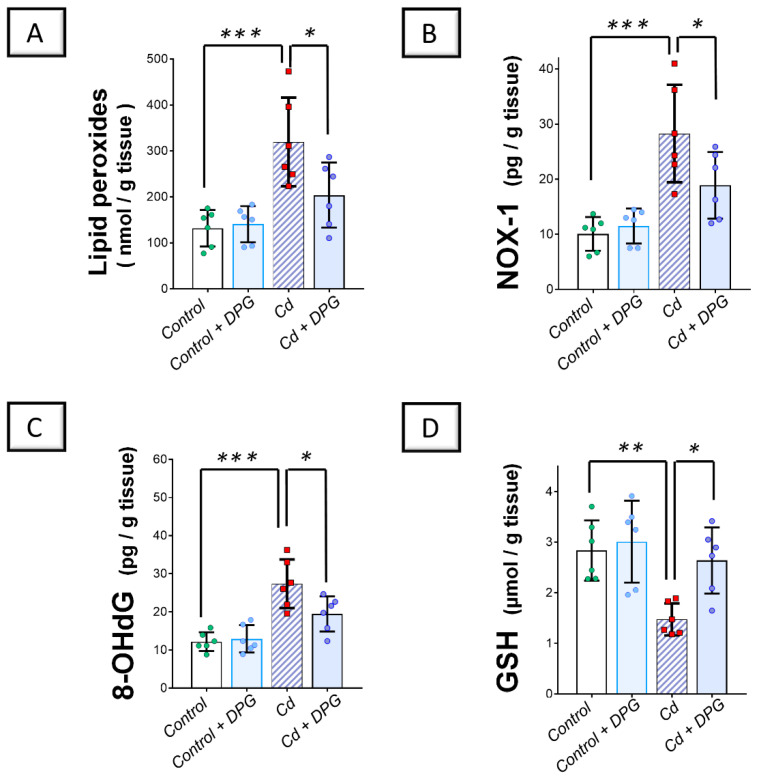
Dapagliflozin improved the Cd-triggered renal oxidative milieu in rats. These effects were proven by the reduced levels of renal lipid peroxides (**A**), NOX-1 (**B**), and 8-OHdG (**C**), alongside an augmented content of renal GSH (**D**), compared to the Cd group. For *n* = 6, the graph bars represent the mean ± standard deviation. Statistical significance was described by * *p* < 0.05, ** *p* < 0.01, or *** *p* < 0.001. Cd, cadmium chloride; DPG, dapagliflozin.

Examination of the renal oxidant–antioxidant milieu revealed that Cd significantly elevated the renal levels (*p* < 0.001) of lipid peroxides, NADPH oxidase 1 (NOX-1), and 8-hydroxy-deoxyguanosine (8-OHdG) pro-oxidant markers by 142.4%, 180.7%, and 124.7%, respectively, versus the control group (Figure 4A–C). Moreover, Cd significantly lowered the reduced glutathione (GSH; *p* < 0.01) by 48% in the renal tissues of animals (Figure 4D). Co-administration of DPG with Cd afforded a significant reduction (*p* < 0.05) in renal lipid peroxides, NOX-1, and 8-OHdG levels by 36.2%, 33.2%, and 28.9%, respectively. Likewise, DPG and Cd co-administration elicited a significant elevation in renal GSH levels (*p* < 0.05) by 78.9% in comparison to the Cd group.

**Figure 5:**
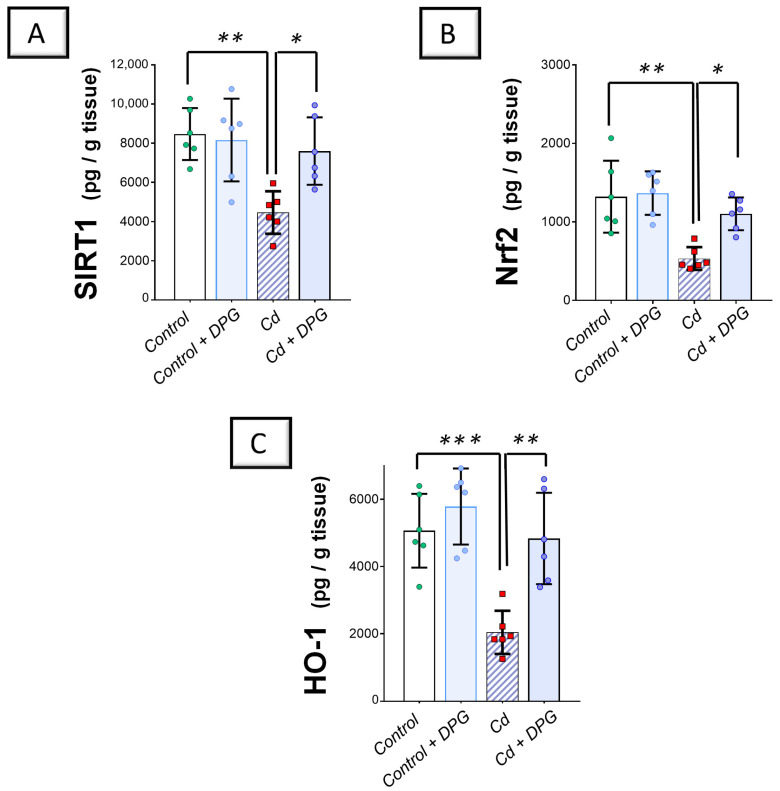
Dapagliflozin counteracted Cd-triggered inhibition in the SIRT1/Nrf2/HO-1 axis of rats. These effects were proven by the augmented levels of renal SIRT1 (**A**), Nrf2 (**B**), and HO-1 (**C**) compared to the Cd group. For *n* = 6, the graph bars represent the mean ± standard deviation. Statistical significance was described by * *p* < 0.05, ** *p* < 0.01, or *** *p* < 0.001. Cd, cadmium chloride; DPG, dapagliflozin.

In terms of the antioxidant SIRT1/Nrf2/heme oxygenase 1 (HO-1) axis, Cd significantly reduced the renal levels of SIRT1 (*p* < 0.01), Nrf2 (*p* < 0.01), and HO-1 (*p* < 0.001) by 47.3%, 59.7%, and 58.4%, respectively, versus the control group (Figure 5A–C). Co-administration of DPG with Cd afforded a significant augmentation in the renal levels of SIRT1 (*p* < 0.05), Nrf2 (*p* < 0.05), and HO-1 (*p* < 0.01) by 70.3%, 106.9%, and 136.7%, respectively. Collectively, these findings advocate the notion that the suppression of renal pro-oxidant events and stimulation of the renal SIRT1/Nrf2/HO-1 axis, at least partly, contribute to the improvement of Cd-triggered nephrotoxicity.

### 2.4. Cadmium-Induced Renal Pro-Apoptotic Machinery Was Diminished by Dapagliflozin in Rats

**Figure 6:**
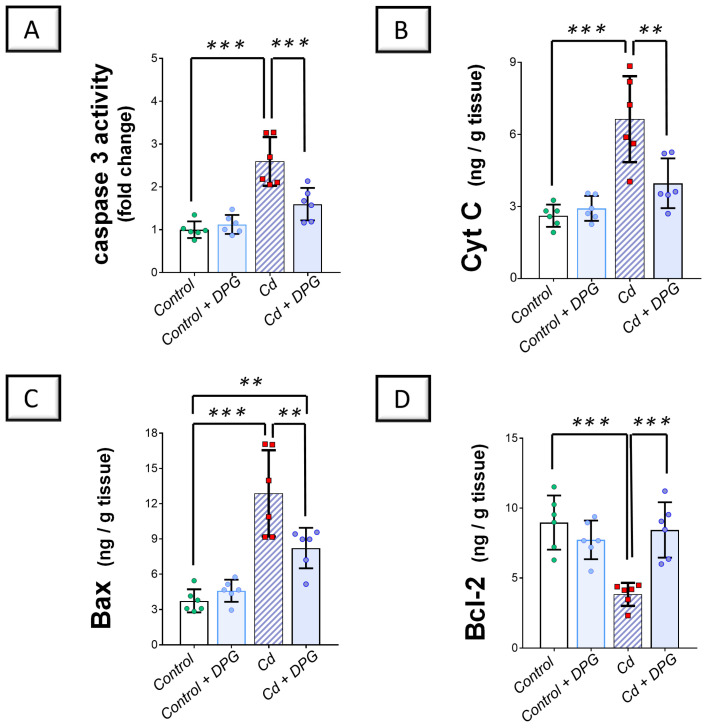
Dapagliflozin counteracted Cd-triggered renal pro-apoptotic events in rats. These effects were proven by the reduced activity of renal caspase 3 (**A**), alongside the levels of Cyt C (**B**), and Bax (**C**), compared to the Cd group. Moreover, DPG augmented renal Bcl-2 protein expression (**D**). For *n* = 6, the graph bars represent the mean ± standard deviation. Statistical significance was described by ** *p* < 0.01 or *** *p* < 0.001. Cd, cadmium chloride; DPG, dapagliflozin.

Exploration of the renal pro-apoptotic machinery revealed that Cd significantly elevated renal caspase 3 activity (*p* < 0.001) alongside the levels of cytochrome C (Cyt C; *p* < 0.001) and Bcl-2 associated-x protein (Bax; *p* < 0.001) as pro-apoptotic markers by 159.4%, 153.2%, and 245.5%, respectively, versus the control group (Figure 6A–C). Moreover, Cd significantly downregulated B-cell lymphoma 2 (Bcl-2) protein expression (*p* < 0.001) by 57.3% in the renal tissues of animals (Figure 6D). Co-administration of DPG with Cd afforded a significant reduction in renal caspase 3 (*p* < 0.001), Cyt C (*p* < 0.01), and Bax (*p* < 0.01) by 38.5%, 40.2%, and 26.2%, respectively. Likewise, DPG and Cd co-administration elicited a significant elevation in Bcl-2 protein expression (*p* < 0.001) by 120.4% in comparison to the Cd group. Collectively, these findings advocate the notion that the suppression of the renal pro-apoptotic machinery, at least partly, contributes to the improvement of Cd-triggered nephrotoxicity.

### 2.5. Cadmium-Induced Impairment in Renal Autophagy Response Was Rescued by Dapagliflozin in Rats

**Figure 7:**
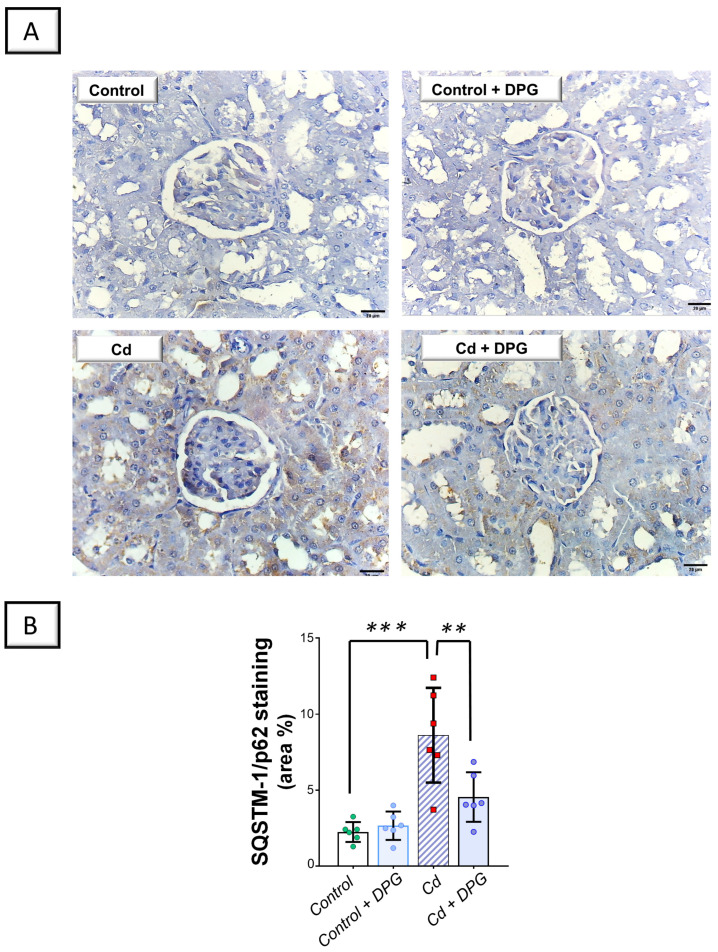
Dapagliflozin counteracted the Cd-triggered renal autophagy blockade in rats. This effect was proven by the lowered levels of SQSTM-1/p62. (**A**) Representative immunohistochemical images of renal SQSTM-1/p62 staining (scale bar: 20 µm). (**B**) SQSTM-1/p62 protein quantification displaying area percentage (calculated using six non-overlapping microscopic fields). For *n* = 6, the graph bars represent the mean ± standard deviation. Statistical significance was described by ** *p* < 0.01 or *** *p* < 0.001. Cd, cadmium chloride; DPG, dapagliflozin.

**Figure 8:**
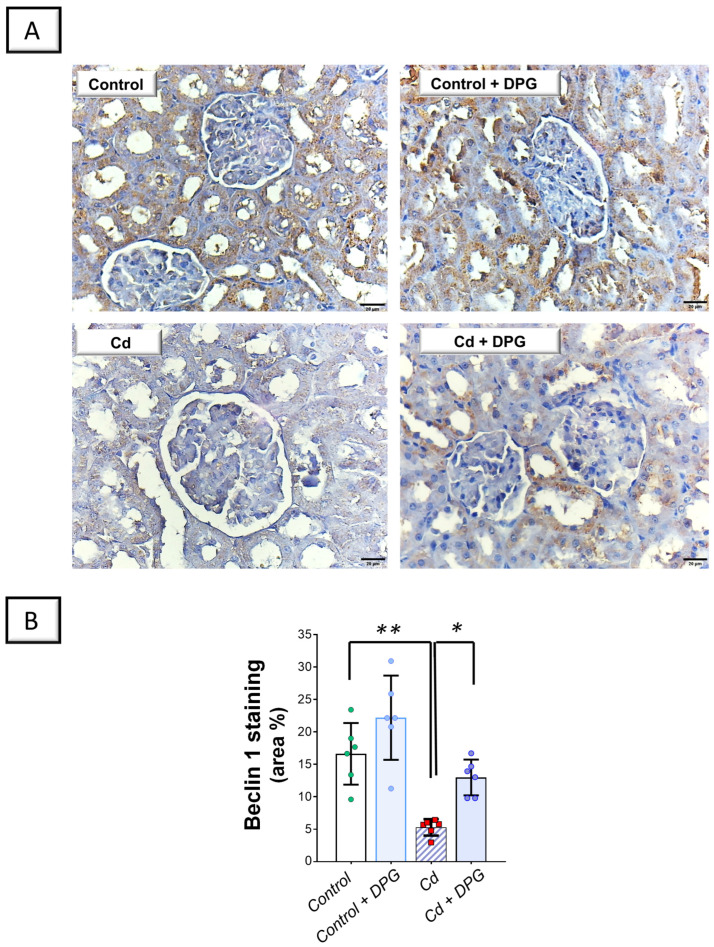
Dapagliflozin reversed Cd-triggered renal Beclin 1 downregulation in rats. (**A**) Representative immunohistochemical images of renal Beclin 1 staining (scale bar: 20 µm). (**B**) Beclin 1 protein quantification displaying area percentage (calculated using six non-overlapping microscopic fields). For *n* = 6, the graph bars represent the mean ± standard deviation. Statistical significance was described by * *p* < 0.05 or ** *p* < 0.01. Cd, cadmium chloride; DPG, dapagliflozin.

Examination of renal autophagy events revealed that Cd significantly elevated the renal levels of sequestosome-1/protein 62 (SQSTM-1/p62; *p* < 0.001) by 284.7%, as a marker of defective autophagy flux, versus the control group (Figure 7B). In tandem, Cd significantly lowered Beclin 1 (*p* < 0.01) by 68.2% in the renal tissues of animals (Figure 8B). Co-administration of DPG with Cd afforded a significant reduction in the renal level of SQSTM-1/p62 (*p* < 0.01) by 47.3% in comparison to the Cd group. In the same regard, DPG and Cd co-administration elicited a significant elevation in Beclin 1 level (*p* < 0.05) by 145.6%. Collectively, these findings advocate the notion that the promotion of the renal pro-autophagy response, at least partly, contributes to the improvement of Cd-triggered nephrotoxicity.

### 2.6. Cadmium-Induced Inhibition of the Renal AMPK/mTOR Cascade Was Counteracted by Dapagliflozin in Rats

**Figure 9:**
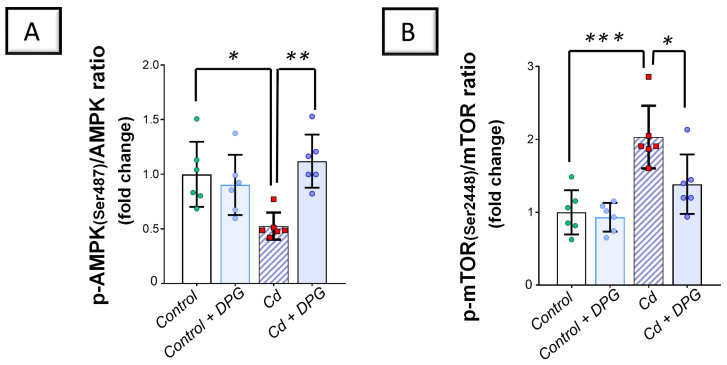
Dapagliflozin counteracted the Cd-triggered inhibition of the renal AMPK/mTOR pathway. These effects were proven by the augmented p-AMPK(Ser487)/total AMPK levels (**A**), together with the diminished levels of p-mTOR(Ser2448)/total mTOR (**B**). For *n* = 6, the graph bars represent the mean ± standard deviation. Statistical significance was described by * *p* < 0.05, ** *p* < 0.01, or *** *p* < 0.001. Cd, cadmium chloride; DPG, dapagliflozin.

Exploration of the renal AMP-activated protein kinase (AMPK)/mechanistic target of rapamycin (mTOR) pathway revealed that Cd significantly lowered renal levels of phosphorylated AMPK/total AMPK (*p* < 0.05) by 47.4% versus the control group, denoting the inhibition of this pathway (Figure 9A). In agreement with this finding, Cd significantly elevated the levels of phosphorylated mTOR/total mTOR (*p* < 0.001) by 103.2% in the renal tissues of animals (Figure 9B). Co-administration of DPG with Cd afforded a significant elevation in the levels of phosphorylated AMPK/total AMPK (*p* < 0.01) by 112.9% in comparison to the Cd group. In the same regard, DPG and Cd co-administration elicited a significant reduction in the levels of phosphorylated mTOR/total mTOR (*p* < 0.05) by 31.8%. Collectively, these findings advocate the notion that stimulation of the renal AMPK/mTOR pathway, at least partly, contributes to eliminating damaged organelles and misfolded proteins from kidney tissues.

## 3. Discussion

**Figure 10:**
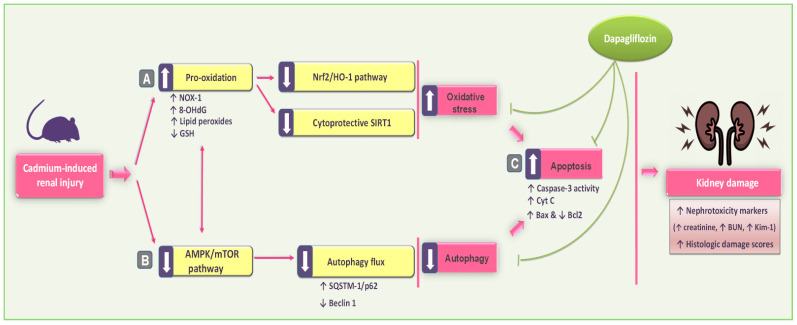
An illustration of dapagliflozin’s beneficial actions in improving Cd-induced renal outcomes in rats. In reference to the present findings, dapagliflozin attenuated renal pathological and functional disturbances in vivo by (**A**) antioxidant actions and stimulation of the renal SIRT1/Nrf2/HO-1 pathway; (**B**) rescue of renal autophagy dysfunction with AMPK/mTOR stimulation; and (**C**) inhibition of renal pro-apoptotic events, thereby promoting cellular survival. In this figure, there are two types of arrows: solid arrows representing activation and blunt arrows representing inhibition.

The current study demonstrates that DPG significantly improved Cd-induced renal pathological and functional disturbances in vivo in rats. These renoprotective effects are attributed to DPG’s multifaceted mechanisms, including antioxidant, antiapoptotic, and autophagy-stimulating features, driven by the activation of the SIRT1/Nrf2/HO-1 and AMPK/mTOR signaling pathways (Figure 10). Notably, the interplay among the SIRT1/Nrf2/HO-1, AMPK/mTOR, autophagy, and apoptotic pathways reinforces the efficacy of DPG in alleviating Cd-induced renal injury. Activation of the SIRT1/Nrf2 pathway enhances antioxidant defenses and promotes autophagic clearance of ROS and damaged organelles [9]. Additionally, AMPK activation further supports autophagy and enhances mitochondrial function, whereas inhibition of apoptosis prevents the death of renal cells [13]. These integrated mechanisms collectively support the renoprotective efficacy of DPG.

Cd-induced renal toxicity has been widely linked to oxidative stress as a central mechanism [6,7,8]. This is primarily due to the excessive generation of reactive oxygen species (ROS) and mitochondrial dysfunction. Additional mechanisms include the disruption of antioxidant defenses through thiol group deactivation and the interference with essential metal ions like selenium (Se) and zinc (Zn), crucial for antioxidant enzymes such as glutathione peroxidase and superoxide dismutase [12,14]. In agreement with these findings, the current results indicated excessive renal pro-oxidant events marked by elevated levels of lipid peroxides, NOX-1, and 8-OHdG, as well as curtailment of the SIRT1/Nrf2/HO-1 pathway. DPG administration counteracted these effects, likely through its potent antioxidant properties by stimulating the SIRT1/Nrf2/HO-1 axis. Consistently, DPG’s antioxidant properties have been previously shown to alleviate gentamicin-induced nephrotoxicity [27], cardiac ischemia/reperfusion injury [28], colitis [25], and rotenone-induced PD [25]. Regarding the SIRT1/Nrf2 pathway, SIRT1 serves as a NAD-dependent histone deacetylase that activates Nrf2 by prompting its nuclear translocation as well as its transcriptional activity. In turn, this promotes the activation of several antioxidant signals downstream of Nrf2, such as HO-1, events that favor renal outcomes in kidney injury models [5]. In several kidney pathology models, SIRT1/Nrf2 pathway activation has also been reported to counteract oxidative stress and apoptosis [5,9]. Activation of the SIRT1/Nrf2/HO-1 pathway not only enhances the antioxidant defense system but also fosters the crosstalk with autophagy pathways, as demonstrated by previous reports confirming its protective role in kidney injury models. In this regard, SIRT1 has been shown to suppress mTOR phosphorylation, resulting in the stimulation of AMPK/mTOR-mediated autophagy with consequent disposal of damaged mitochondria [9]. Moreover, several studies have revealed that Nrf2 upregulates the expression of numerous autophagy-related genes, including *Atg3*, *Atg5*, and *Atg7* [36]. Supporting this, Komatsu et al. [37] have demonstrated that SQSTM1/p62 inhibits Keap1 activity, thereby leading to enhanced Nrf2 signaling. Notably, the elimination of damaged mitochondria and ROS by autophagy has been reported to mitigate the pro-oxidant response in an in vitro model of Cd-evoked proximal tubular cell cytotoxicity [1,4] and Cd-prompted renal injury in vivo [14].

A significant impairment in autophagy, a cellular process critical for removing damaged organelles and proteins, is observed in Cd-induced nephrotoxicity [6,12]. In this regard, Cd exposure impairs autophagy through two distinct mechanisms: disruption of calcium-dependent lysosomal fusion with autophagosomes, as well as damage to lysosomal membranes [38]. In agreement with these data, the current study revealed that Cd increased SQSTM1/p62 accumulation and inhibited the AMPK/mTOR pathway in rats denoting impaired renal autophagy. In contrast to some previous studies that examined the effects of heavy metals, including cadmium, on the autophagy process, the observed phosphorylation/activation of mTOR by cadmium in the current study seems contradictory [20,39]. The explanation of the disparity may be related to cadmium doses, animal species, administration routes, exposure durations, and the severity of renal damage [17,20,40,41]. In renal damage models, several renal pathologies in rodents have also been associated with impaired autophagy events, including cisplatin-[19] and calcium oxalate-[18] evoked renal damage. Notably, there is a significant discrepancy between the current findings of Cd-induced impaired autophagy and those reported by Li et al. [20] that characterized an overactive autophagy in the kidney of Cd-intoxicated Sprague–Dawley rats. The disparity in Cd-induced autophagy responses observed across studies—ranging from overactivation to suppression—may depend on differences in Cd-exposure span and renal injury severity. In this regard, it has been reported that mild cellular damage triggers autophagy activation to eliminate ROS and damaged organelles from the cells. On the other hand, severe cellular damage results in excessive autophagy and cell death [17,41].

In a novel finding, DPG effectively restored autophagic events in the present study by stimulating the AMPK/mTOR pathway in vivo in rats. In agreement with our findings, substantial evidence supports the role of autophagy stimulation in mitigating experimental renal injury [7]. The regulation of autophagy and mitochondrial dynamics is largely mediated by AMPK. Activation of SIRT1 and inhibition of mTOR serve as key important stimulators of AMPK, leading to autophagy stimulation [42]. In the same regard, selective inactivation of mTOR has been reported to confer significant renal protection [17]. Mechanistically, activation of autophagy favors cell survival by prompting the recycling of toxic protein aggregates and damaged mitochondria [10]. Consequently, therapeutic agents that enhance autophagy have been shown to alleviate nephrotoxicity [7,10]. In line with these data, DPG has been reported to protect against organ damage due to its pro-autophagic properties. In a rat model of diabetic nephropathy, DPG monotherapy or in combination with metformin has attenuated renal histomorphological aberrations and renal function outcomes through stimulation of autophagy that was evidenced by the diminished accumulation of SQSTM1/p62. Moreover, DPG has been proven to activate the SIRT1/AMPK/mTOR axis in the renal tissue of diabetic rats by lowering p-mTOR levels together with elevating p-AMPK and SIRT1 cytoprotective signals [13]. Another study in lipopolysaccharide-evoked lung injury has demonstrated DPG’s efficacy in dampening pulmonary pathology by modulating AMPK/NFκB signaling in rats [43]. Moreover, DPG’s pro-autophagic properties have been shown to alleviate several pathologies, such as myocardial ischemia/reperfusion injury [44], diabetic cardiac failure [45], testicular damage [29], Alzheimer-like manifestations [30], and cadmium-evoked cognitive decline [46].

Excessive ROS generated during Cd exposure not only impair antioxidant defenses but also trigger apoptosis, primarily via the mitochondrial intrinsic pathway [15]. In this regard, the mitochondrial intrinsic pathway has been viewed as one of the major mechanisms for triggering apoptosis in kidney cells [15] by decreasing Bcl-2 expression and increasing Bax expression [13]. During apoptosis by the mitochondrial intrinsic pathway, cytochrome C leaks into the cytoplasm, which, in turn, triggers activation of caspases that play an important role as cysteine proteases. Caspase 3 has been recognized as a major mediator in cellular degradation and DNA fragmentation, which leads to cell death [4,32]. Of note, Bcl-2 has been reported to regulate the interplay between autophagy and apoptosis [1,15]. Consistent with these data, our findings demonstrated that Cd enhanced the apoptosis of renal cells as determined by the upregulation of Bax and activation of caspase 3. Interestingly, the renal apoptotic machinery was inhibited by DPG, which counteracted these molecular events. DPG’s anti-apoptotic effects are well-supported in the literature. In a rat model of diabetic nephropathy, DPG has reduced renal apoptosis by downregulating Cyt C and Bax, resulting in improved renal functions [13]. Similarly, curbing cardiac apoptosis has mediated the beneficial outcomes of DPG in an experimental model of cardiac dysfunction [47]. Moreover, DPG has also been shown to dampen apoptosis in the retina of diabetic mice and retinal microvascular endothelial cells [48]. The interplay between apoptosis and autophagy is notable, as Beclin 1 and SQSTM1/p62 function as markers that regulate these processes. By alleviating the Cd-induced imbalance between autophagy and apoptosis [13,43,44], DPG contributes to renal protection.

## 4. Materials and Methods

### 4.1. Animals

Wistar albino rats (180–220 g weight; 8–10 weeks old) were used in this experiment. In the animal facility, rats were housed under controlled conditions including a 21–24 °C temperature, 12 h alternating light/dark cycle, and 50% humidity. An acclimatization period of one week preceded the start of the experimental protocol. Taif University’s Research Ethical Committee approved this study’s protocol according to its regulations (Ethical Reference No: 45-242). During the conduct of the study, animal handling was applied as guided by the Laboratory Animal Guide (National Institutes of Health, Bethesda, MD, USA).

### 4.2. Chemicals and Drugs

Sigma-Aldrich (Carlsbad, CA, USA) supplied the cadmium chloride (CdCl_2_). The pharmaceutical company AstraZeneca (Cairo, Egypt) provided the dapagliflozin. This work involved the use of high-quality chemicals for all assays.

### 4.3. Study Design

Four experimental groups of six animals were randomly distributed by a blinded technician: (**1**) **Control group**: an oral gavage of saline was received daily by rats for 2 months. Moreover, an oral gavage of 0.5% carboxymethylcellulose sodium (CMC) was administered daily to rats for the same period. Between the two doses, there was a two-hour interval. (**2**) **Control + DPG group**: an oral gavage of saline was received daily by rats for 2 months. Moreover, an oral gavage of dapagliflozin was administered daily (1 mg/kg/day; in CMC) to rats for the same period. Between the two doses, there was a two-hour interval. (**3**) **Cd group**: an oral gavage of CdCl_2_ (5 mg/kg/day in saline) was received daily by rats for 2 months. Moreover, an oral gavage of CMC was administered daily to rats for the same period. Between the two doses, there was a two-hour interval. (**4**) **Cd + DPG group**: an oral gavage of CdCl_2_ (5 mg/kg/day in saline) was received daily by rats for 2 months. Moreover, an oral gavage of dapagliflozin was administered daily (1 mg/kg/day; in CMC) to rats for the same period. Between the two doses, there was a two-hour interval.

The DPG dose regimen is consistent with the dose commonly used in clinical practice based on the human equivalent dose (HED) approach [49]. Moreover, the DPG dose selection is based on previous studies that signified its competence in improving experimental inflammatory bowel disease [25], gentamicin-induced nephrotoxicity [27], cardiac ischemia/reperfusion injury [28], myocardial infarction [26], Parkinson’s disease [50], and PTZ-triggered epilepsy [51]. In the same regard, the current experimental protocol is consistent with previous publications [12,14,16,20].

### 4.4. Specimen Collection

After the experimental study was completed, all rats were anesthetized with thiopental (30 mg/kg; i.p.). Intracardiac punctures were used to collect blood samples. Then, kidneys were immediately collected, decapsulated, and weighed after an animal sacrifice was performed under anesthesia. After standing at 37 °C for 0.5 h, blood sample centrifugation was conducted (1000× *g*; 10 min) for serum collection. Morphological analysis and immunohistochemistry were conducted on one kidney from three randomly selected animals per group after immersion in a neutral tissue fixative. For biochemical analysis, the other kidney was placed in liquid nitrogen and stored at −80 °C. To this end, kidney tissue was homogenized in RIPA buffer. Homogenate centrifugation was applied (1000× *g*; 10 min) for supernatant isolation.

### 4.5. Kidney Function Tests

A commercial ELISA kit was used to measure renal KIM-1 (Elabscience, Wuhan, China), according to the instructions provided by the vendor. Determination of the colored product’s intensity in the reaction mixture was applied at 450 nm (SpectraMax iD5, Molecular Devices, San Jose, CA, USA). Moreover, colorimetric assays were performed to measure urea and creatinine levels in the serum.

### 4.6. Renal Cadmium Content

In this study, the amount of cadmium in rat kidney tissue was quantified following the procedure previously described [52]. In summary, kidney samples were weighed and digested in 1 M nitric acid at 150 °C for two hours. A graphite furnace atomic absorption spectrophotometer (Perkin-Elmer 3100, Norfolk, CT, USA) was used to measure cadmium levels at 228.8 nm.

### 4.7. Histopathology

As previously outlined [53], kidneys from three randomly selected animals per experimental group were fixed overnight in neutral tissue fixative, then transferred to PBS and embedded in paraffin. An interval of 160 µm was established for sectioning, and a random point on the caudal pole was selected for the start of the sectioning. The upper pole, middle portion, and lower pole of the kidney were randomly selected to improve the representation of kidney tissue as a whole. The staining of paraffin-embedded specimens with hematoxylin and eosin (H-E) was performed to assess the general histological condition of the specimens. Under a light microscope (bright-field; Model MX5200L, Meiji Techno Co., Saitama, Japan), both the glomerular and tubular parts of the slides were examined for morphological aberrations. While capturing and analyzing images, the identity of the specimen was kept anonymous to avoid bias. From each experimental group, at least six random fields were examined as part of the histopathology analysis.

Endothelial/glomerular/tubular/interstitial (EGTI) damage scoring was applied to quantify the severity of kidney histopathological damage, as previously detailed [53]. In this system, the total damage score ranges from 0, indicating a lack of renal histopathological damage, to 14, revealing severe renal damage. The total EGTI scoring was based on four types of histological damage that were quantified: endothelial (0 to 3 scores), glomerular (0 to 3 scores), tubular (0 to 4 scores), and interstitial (0 to 4 scores) damage. Finally, the scores of the 4 components were added together to calculate the EGTI damage scores.

### 4.8. Investigation of Renal Redox Milieu and Serum Glucose

The lipid peroxide content, expressed as malondialdehyde, was measured using a thiobarbituric-acid-based colorimetric assay as previously described [54]. Determination of the colored product’s intensity in the reaction mixture was applied at 535 nm (SpectraMax iD5, Molecular Devices, San Jose, CA, USA). The contents of 8-hydroxy-deoxyguanosine (8-OHdG; AFG Bioscience, Northbrook. IL, USA) and NADPH oxidase 1 (NOX-1; AFG Bioscience, Northbrook, IL, USA) in the renal tissue were assayed using specific ELISA kits, according to the guidance provided by the supplier. With the aid of a UV/visible ELISA plate reader (SpectraMax iD5, Molecular Devices, San Jose, CA, USA), the determination of the colored product’s intensity in the reaction mixture was conducted at 450 nm. A reduced glutathione (GSH) measurement was conducted according to the colorimetric assay established by Beutler et al. [55] with modification [56]. Determination of the colored product’s intensity in the reaction mixture was conducted at 412 nm (SpectraMax iD5, Molecular Devices, San Jose, CA, USA).

The contents of Nrf2 (SunLong Biotech, Zhejiang, China), HO-1 (Elabscience, Wuhan, China), and SIRT1 (Aviva System Biology, San Diego, CA, USA) in the renal tissue were assayed using specific ELISA kits, according to the guidance provided by the supplier. With the aid of a UV/visible ELISA plate reader (SpectraMax iD5, Molecular Devices, San Jose, CA, USA), the determination of the colored product’s intensity in the reaction mixture was applied at 450 nm. For the Nrf2 assay, a Cayman kit was used for the extraction of nuclear total proteins (Cayman Chemical, Ann Arbor, MA, USA). A colorimetric kit was utilized for quantifying serum glucose (HUMAN Diagnostics, Wiesbaden, Germany), as guided by the supplier. Determination of the colored product’s intensity in the reaction mixture was applied at 500 nm (SpectraMax iD5, Molecular Devices, San Jose, CA, USA).

### 4.9. Renal Apoptosis

The contents of Bcl-2 associated-x protein (Bax; MyBioSource, San Diego, CA, USA), cytochrome C (Cyt C; Elabscience Biotechnology, Houston, TX, USA), and B cell lymphoma 2 protein (Bcl2; MyBioSource, San Diego, CA, USA) in the renal tissue were assayed using specific ELISA kits, according to the instructions provided by the manufacturer (San Diego, CA, USA). With the aid of a UV/visible ELISA plate reader (SpectraMax iD5, Molecular Devices, San Jose, CA, USA), the determination of the colored product’s intensity in the reaction mixture was applied at 450 nm. A Sigma-Aldrich colorimetric kit was used for the assay of caspase 3 activity, as guided by the supplier (Carlsbad, CA, USA). Determination of the colored product’s intensity in the reaction mixture was applied at 405 nm (SpectraMax iD5, Molecular Devices, San Jose, CA, USA). Following the vendor’s guidance and former studies [14], a fold change was used to calculate the values of caspase 3 activity.

### 4.10. Immunohistochemistry

In each experimental group, 3 kidney samples were randomly selected from 3 rats. Five sagittal kidney sections were mounted on glass slides for the immunostaining protocol. An interval of 160 µm was established for sectioning, and a random point on the caudal pole was selected for the start of the sectioning. The upper pole, middle portion, and lower pole of the kidney were randomly selected in order to improve the representation of kidney tissue as a whole. Deparaffinization with xylene and the rehydration of unstained kidney sections (5 microns) were applied. Then, endogenous peroxidase activity was blocked by incubating them for 30 min with 3% H_2_O_2_. A citrate solution was used for antigen retrieval at 95 °C for 20 min [57]. Aiming to stain sequestosome-1/protein 62 (SQSTM-1/p62) and Beclin 1, section incubation at 4 °C overnight was conducted using the following antibodies: anti-Beclin 1 or anti-SQSTM-1/p62 (1: 100 dilution, Abcam, Cambridge, MA, USA). Section incubation with secondary antibodies tagged with horseradish peroxidase (HRP) was applied for 30 min. As directed by the manufacturer, a detection kit was used to determine the extent of positive areas in the samples (HRP Envision kit, DAKO, Santa Clara, CA, USA) under a light microscope (Model MX5200L, Meiji Techno Co., Saitama, Japan). To visualize immunostaining, we used diaminobenzidine (DAB). The total number of positive areas on each slide was calculated by randomly selecting six fields from the kidney cortex and medulla. Using Fiji ImageJ^®^ software (1.51r version), the area of brown immunostaining was measured, and image analysis (NIH, Bethesda, MD, USA) was performed. While capturing and analyzing images, the identity of the specimen was kept anonymous to inhibit any bias.

### 4.11. Determination of Renal Autophagy Events

The content of total AMPK and p-AMPK (Ser487) in the renal tissue was measured using an ELISA kit provided by RayBiotech, according to the instructions provided by the manufacturer (Norcross, GA, USA). In addition to determining the phosphorylated form (Ser487) of the target protein (half of each 96-well plate), the kit was also capable of determining the non-phosphorylated form of AMPK. With the aid of a UV/visible ELISA plate reader (SpectraMax iD5, Molecular Devices, San Jose, CA, USA), the determination of the colored product’s intensity in the reaction mixture was conducted at 450 nm. The content of total mTOR and p-mTOR(Ser2448) in the renal tissue was measured using ELISA kits provided by Cell Signaling Technology, according to the instructions provided by the manufacturer (Danvers, MA, USA). With the aid of a UV/visible ELISA plate reader (SpectraMax iD5, Molecular Devices, San Jose, CA, USA), the determination of the colored product’s intensity in the reaction mixture was applied at 450 nm. As guided by the supplier as well as former studies [29], a fold change was used to calculate the ratios for p-AMPK (Ser487)/total AMPK as well as p-mTOR (Ser2448)/total mTOR.

### 4.12. Statistics

Statistical analyses were conducted to evaluate the effects of treatments on the biochemical, histological, and molecular endpoints. In this study, the independent variable was the experimental groups, which included the control, DPG-only, Cd-only, and Cd + DPG. The dependent variables measured included biochemical markers such as serum creatinine, urea, and KIM-1. Histopathological parameters, such as EGTI scores, were also analyzed. In addition, the molecular markers related to the SIRT1/Nrf2/HO-1 pathway, the AMPK/mTOR autophagic pathway, oxidative stress markers (lipid peroxides, NOX-1, 8-OHdG, and GSH), and apoptotic markers (e.g., Bax, Cyt C, and caspase 3 activity) were analyzed.

Parametric values were shown as the mean ± standard deviation, whereas non-parametric values were presented as the median with the interquartile range. The parametric variables were analyzed using a one-way ANOVA to compare the results between groups. To identify specific differences between the groups, post hoc comparisons were carried out using a Bonferroni test. An analysis of the data not following a normal distribution (non-parametric data) was performed using a Kruskal–Wallis test to assess overall group differences, followed by a Dunn’s post hoc test to identify individual group differences. To be considered statistically significant, results had to have a *p*-value of less than 0.05.

## 5. Conclusions

The current study reveals that DPG effectively mitigates Cd-induced renal dysfunction and associated pathological changes. These nephroprotective effects were mediated by activating AMPK/mTOR, stimulating renal autophagy, and enhancing the SIRT1/Nrf2/HO-1 antioxidant pathway. Additionally, DPG reduced renal apoptosis and oxidative stress markers, demonstrating its multifaceted protective properties. Notably, further studies are warranted to examine the detailed pharmacokinetics of cadmium—in serum and kidney—in response to multiple doses of dapagliflozin. These studies will provide more insight into the possible interactions between dapagliflozin and cadmium. Additionally, further in vitro studies utilizing relevant kidney cell lines are required to corroborate the current findings.

## Data Availability

Data are contained within the article.

## References

[1] Wang X.Y., Yang H., Wang M.G., Yang D.B., Wang Z.Y., Wang L. (2017). Trehalose protects against cadmium-induced cytotoxicity in primary rat proximal tubular cells via inhibiting apoptosis and restoring autophagic flux. Cell Death Dis..

[2] Prozialeck W.C., Edwards J.R. (2012). Mechanisms of cadmium-induced proximal tubule injury: New insights with implications for biomonitoring and therapeutic interventions. J. Pharmacol. Exp. Ther..

[3] Klaassen C.D., Liu J., Diwan B.A. (2009). Metallothionein protection of cadmium toxicity. Toxicol. Appl. Pharmacol..

[4] Wang L.Y., Fan R.F., Yang D.B., Zhang D., Wang L. (2019). Puerarin reverses cadmium-induced lysosomal dysfunction in primary rat proximal tubular cells via inhibiting Nrf2 pathway. Biochem. Pharmacol..

[5] Shi S., Lei S., Tang C., Wang K., Xia Z. (2019). Melatonin attenuates acute kidney ischemia/reperfusion injury in diabetic rats by activation of the SIRT1/Nrf2/HO-1 signaling pathway. Biosci. Rep..

[6] Gong Z.-G., Zhao Y., Wang Z.-Y., Fan R.-F., Liu Z.-P., Wang L. (2022). Epigenetic regulator BRD4 is involved in cadmium-induced acute kidney injury via contributing to lysosomal dysfunction, autophagy blockade and oxidative stress. J. Hazard. Mater..

[7] Ma Y., Su Q., Yue C., Zou H., Zhu J., Zhao H., Song R., Liu Z. (2022). The Effect of Oxidative Stress-Induced Autophagy by Cadmium Exposure in Kidney, Liver, and Bone Damage, and Neurotoxicity. Int. J. Mol. Sci..

[8] Zhang K., Dong W., Li J., Gong Z., Liu W., He S., Zou H., Song R., Liu G., Liu Z. (2022). Honokiol Antagonizes Cadmium-Induced Nephrotoxicity in Quail by Alleviating Autophagy Dysfunction, Apoptosis and Mitochondrial UPR Inhibition with Its Antioxidant Properties. Life.

[9] Guan Y., Hao C.M. (2016). SIRT1 and Kidney Function. Kidney Dis..

[10] Thévenod F., Lee W.-K. (2015). Live and let die: Roles of autophagy in cadmium nephrotoxicity. Toxics.

[11] Lee H.Y., Oh S.H. (2021). Autophagy-mediated cytoplasmic accumulation of p53 leads to apoptosis through DRAM-BAX in cadmium-exposed human proximal tubular cells. Biochem. Biophys. Res. Commun..

[12] Fan R.F., Tang K.K., Wang Z.Y., Wang L. (2021). Persistent activation of Nrf2 promotes a vicious cycle of oxidative stress and autophagy inhibition in cadmium-induced kidney injury. Toxicology.

[13] Jaikumkao K., Thongnak L., Htun K.T., Pengrattanachot N., Phengpol N., Sutthasupha P., Promsan S., Montha N., Sriburee S., Kothan S. (2024). Dapagliflozin and metformin in combination ameliorates diabetic nephropathy by suppressing oxidative stress, inflammation, and apoptosis and activating autophagy in diabetic rats. Biochim. Biophys. Acta Mol. Basis Dis..

[14] Arab H.H., Ashour A.M., Eid A.H., Arafa E.A., Al Khabbaz H.J., Abd El-Aal S.A. (2022). Targeting oxidative stress, apoptosis, and autophagy by galangin mitigates cadmium-induced renal damage: Role of SIRT1/Nrf2 and AMPK/mTOR pathways. Life Sci..

[15] Marino G., Niso-Santano M., Baehrecke E.H., Kroemer G. (2014). Self-consumption: The interplay of autophagy and apoptosis. Nat. Rev. Mol. Cell Biol..

[16] Chen J., Lai W., Deng Y., Liu M., Dong M., Liu Z., Wang T., Li X., Zhao Z., Yin X. (2021). MicroRNA-363-3p promotes apoptosis in response to cadmium-induced renal injury by down-regulating phosphoinositide 3-kinase expression. Toxicol. Lett..

[17] Kaushal G.P., Chandrashekar K., Juncos L.A., Shah S.V. (2020). Autophagy Function and Regulation in Kidney Disease. Biomolecules.

[18] Wu Y., Xun Y., Zhang J., Hu H., Qin B., Wang T., Wang S., Li C., Lu Y. (2021). Resveratrol Attenuates Oxalate-Induced Renal Oxidative Injury and Calcium Oxalate Crystal Deposition by Regulating TFEB-Induced Autophagy Pathway. Front. Cell Dev. Biol..

[19] Qu X., Gao H., Tao L., Zhang Y., Zhai J., Song Y., Zhang S. (2018). Autophagy inhibition-enhanced assembly of the NLRP3 inflammasome is associated with cisplatin-induced acute injury to the liver and kidneys in rats. J. Biochem. Mol. Toxicol..

[20] Li J.R., Ou Y.C., Wu C.C., Wang J.D., Lin S.Y., Wang Y.Y., Chen W.Y., Liao S.L., Chen C.J. (2020). Endoplasmic reticulum stress and autophagy contributed to cadmium nephrotoxicity in HK-2 cells and Sprague-Dawley rats. Food Chem. Toxicol..

[21] Nashar K., Khalil P. (2022). Clinical Evaluation of Dapagliflozin in the Management of CKD: Focus on Patient Selection and Clinical Perspectives. Int. J. Nephrol. Renovasc. Dis..

[22] Jabbour S., Goldstein B. (2008). Sodium glucose co-transporter 2 inhibitors: Blocking renal tubular reabsorption of glucose to improve glycaemic control in patients with diabetes. Int. J. Clin. Pract..

[23] Tirmenstein M., Dorr T.E., Janovitz E.B., Hagan D., Abell L.M., Onorato J.M., Whaley J.M., Graziano M.J., Reilly T.P. (2013). Nonclinical toxicology assessments support the chronic safety of dapagliflozin, a first-in-class sodium-glucose cotransporter 2 inhibitor. Int. J. Toxicol..

[24] Wright E.M., Loo D.D., Hirayama B.A. (2011). Biology of human sodium glucose transporters. Physiol. Rev..

[25] Arab H.H., Al-Shorbagy M.Y., Saad M.A. (2021). Activation of autophagy and suppression of apoptosis by dapagliflozin attenuates experimental inflammatory bowel disease in rats: Targeting AMPK/mTOR, HMGB1/RAGE and Nrf2/HO-1 pathways. Chem. Biol. Interact..

[26] Lee T.-M., Chang N.-C., Lin S.-Z. (2017). Dapagliflozin, a selective SGLT2 Inhibitor, attenuated cardiac fibrosis by regulating the macrophage polarization via STAT3 signaling in infarcted rat hearts. Free Radic. Biol. Med..

[27] Mohamed D.I., Khairy E., Saad S.S.T., Habib E.K., Hamouda M.A. (2019). Potential protective effects of Dapagliflozin in gentamicin induced nephrotoxicity rat model via modulation of apoptosis associated miRNAs. Gene.

[28] Lahnwong S., Palee S., Apaijai N., Sriwichaiin S., Kerdphoo S., Jaiwongkam T., Chattipakorn S.C., Chattipakorn N. (2020). Acute dapagliflozin administration exerts cardioprotective effects in rats with cardiac ischemia/reperfusion injury. Cardiovasc. Diabetol..

[29] Arab H.H., Fikry E.M., Alsufyani S.E., Ashour A.M., El-Sheikh A.A.K., Darwish H.W., Al-Hossaini A.M., Saad M.A., Al-Shorbagy M.Y., Eid A.H. (2023). Stimulation of Autophagy by Dapagliflozin Mitigates Cadmium-Induced Testicular Dysfunction in Rats: The Role of AMPK/mTOR and SIRT1/Nrf2/HO-1 Pathways. Pharmaceuticals.

[30] Abd Elmaaboud M.A., Estfanous R.S., Atef A., Kabel A.M., Alnemari K.A., Naguib T.M., Alsufyani S.E., Darwish H.W., Arab H.H. (2023). Dapagliflozin/Hesperidin Combination Mitigates Lipopolysaccharide-Induced Alzheimer’s Disease in Rats. Pharmaceuticals.

[31] Li L., Li Q., Huang W., Han Y., Tan H., An M., Xiang Q., Zhou R., Yang L., Cheng Y. (2021). Dapagliflozin Alleviates Hepatic Steatosis by Restoring Autophagy via the AMPK-mTOR Pathway. Front. Pharmacol..

[32] Jaikumkao K., Promsan S., Thongnak L., Swe M.T., Tapanya M., Htun K.T., Kothan S., Intachai N., Lungkaphin A. (2021). Dapagliflozin ameliorates pancreatic injury and activates kidney autophagy by modulating the AMPK/mTOR signaling pathway in obese rats. J. Cell. Physiol..

[33] Yuan Y., Sun M., Jin Z., Zheng C., Ye H., Weng H. (2023). Dapagliflozin ameliorates diabetic renal injury through suppressing the self-perpetuating cycle of inflammation mediated by HMGB1 feedback signaling in the kidney. Eur. J. Pharmacol..

[34] Darwish S.F., Mahmoud A.M.A., Abdel Mageed S.S., Sallam A.M., Oraby M.A. (2023). Dapagliflozin improves early acute kidney injury induced by vancomycin in rats: Insights on activin A/miRNA-21 signaling and FOXO3a expression. Eur. J. Pharmacol..

[35] Dhillon S. (2019). Dapagliflozin: A review in type 2 diabetes. Drugs.

[36] Copple I.M., Lister A., Obeng A.D., Kitteringham N.R., Jenkins R.E., Layfield R., Foster B.J., Goldring C.E., Park B.K. (2010). Physical and functional interaction of sequestosome 1 with Keap1 regulates the Keap1-Nrf2 cell defense pathway. J. Biol. Chem..

[37] Komatsu M., Kurokawa H., Waguri S., Taguchi K., Kobayashi A., Ichimura Y., Sou Y.-S., Ueno I., Sakamoto A., Tong K.I. (2010). The selective autophagy substrate p62 activates the stress responsive transcription factor Nrf2 through inactivation of Keap1. Nat. Cell Biol..

[38] Liu F., Wang X.-Y., Zhou X.-P., Liu Z.-P., Song X.-B., Wang Z.-Y., Wang L. (2017). Cadmium disrupts autophagic flux by inhibiting cytosolic Ca2+-dependent autophagosome-lysosome fusion in primary rat proximal tubular cells. Toxicology.

[39] Huang J., Ma X.T., Xu D.D., Yao B.J., Zhao D.Q., Leng X.Y., Liu J. (2021). Xianling Gubao Capsule Prevents Cadmium-Induced Kidney Injury. BioMed Res. Int..

[40] Avila-Rojas S.H., Lira-Leon A., Aparicio-Trejo O.E., Reyes-Fermin L.M., Pedraza-Chaverri J. (2019). Role of Autophagy on Heavy Metal-Induced Renal Damage and the Protective Effects of Curcumin in Autophagy and Kidney Preservation. Medicina.

[41] Luo S., Rubinsztein D.C. (2010). Apoptosis blocks Beclin 1-dependent autophagosome synthesis: An effect rescued by Bcl-xL. Cell Death Differ..

[42] Juszczak F., Caron N., Mathew A.V., Decleves A.E. (2020). Critical Role for AMPK in Metabolic Disease-Induced Chronic Kidney Disease. Int. J. Mol. Sci..

[43] Abd El-Fattah E.E., Saber S., Mourad A.A., El-Ahwany E., Amin N.A., Cavalu S., Yahya G., Saad A.S., Alsharidah M., Shata A. (2022). The dynamic interplay between AMPK/NFκB signaling and NLRP3 is a new therapeutic target in inflammation: Emerging role of dapagliflozin in overcoming lipopolysaccharide-mediated lung injury. Biomed. Pharmacother..

[44] Yu Y.W., Que J.Q., Liu S., Huang K.Y., Qian L., Weng Y.B., Rong F.N., Wang L., Zhou Y.Y., Xue Y.J. (2021). Sodium-Glucose Co-transporter-2 Inhibitor of Dapagliflozin Attenuates Myocardial Ischemia/Reperfusion Injury by Limiting NLRP3 Inflammasome Activation and Modulating Autophagy. Front. Cardiovasc. Med..

[45] Feng B., Yu P., Yu H., Qian B., Li Y., Sun K., Shi B., Zhang N., Xu G. (2023). Therapeutic effects on the development of heart failure with preserved ejection fraction by the sodium-glucose cotransporter 2 inhibitor dapagliflozin in type 2 diabetes. Diabetol. Metab. Syndr..

[46] Arab H.H., Eid A.H., Alsufyani S.E., Ashour A.M., El-Sheikh A.A.K., Darwish H.W., Sabry F.M. (2023). Targeting Autophagy, Apoptosis, and Oxidative Perturbations with Dapagliflozin Mitigates Cadmium-Induced Cognitive Dysfunction in Rats. Biomedicines.

[47] Liu T., Wu J., Shi S., Cui B., Xiong F., Yang S., Yan M. (2023). Dapagliflozin attenuates cardiac remodeling and dysfunction in rats with β-adrenergic receptor overactivation through restoring calcium handling and suppressing cardiomyocyte apoptosis. Diabetes Vasc. Dis. Res..

[48] Hu Y., Xu Q., Li H., Meng Z., Hao M., Ma X., Lin W., Kuang H. (2022). Dapagliflozin Reduces Apoptosis of Diabetic Retina and Human Retinal Microvascular Endothelial Cells Through ERK1/2/cPLA2/AA/ROS Pathway Independent of Hypoglycemic. Front. Pharmacol..

[49] Freireich E.J., Gehan E., Rall D., Schmidt L., Skipper H. (1966). Quantitative comparison of toxicity of anticancer agents in mouse, rat, hamster, dog, monkey, and man. Cancer Chemother. Rep..

[50] Arab H.H., Safar M.M., Shahin N.N. (2021). Targeting ROS-Dependent AKT/GSK-3beta/NF-kappaB and DJ-1/Nrf2 Pathways by Dapagliflozin Attenuates Neuronal Injury and Motor Dysfunction in Rotenone-Induced Parkinson’s Disease Rat Model. ACS Chem. Neurosci..

[51] Erdogan M.A., Yusuf D., Christy J., Solmaz V., Erdogan A., Taskiran E., Erbas O. (2018). Highly selective SGLT2 inhibitor dapagliflozin reduces seizure activity in pentylenetetrazol-induced murine model of epilepsy. BMC Neurol..

[52] Elkhadragy M.F., Al-Olayan E.M., Al-Amiery A.A., Abdel Moneim A.E. (2018). Protective Effects of Fragaria ananassa Extract Against Cadmium Chloride-Induced Acute Renal Toxicity in Rats. Biol. Trace Elem. Res..

[53] Chavez R., Fraser D.J., Bowen T., Jenkins R.H., Nesargikar P., Pino-Chavez G., Khalid U. (2016). Kidney ischaemia reperfusion injury in the rat: The EGTI scoring system as a valid and reliable tool for histological assessment. J. Histol. Histopathol..

[54] Buege J.A., Aust S.D. (1978). Microsomal lipid peroxidation. Methods Enzymol..

[55] Beutler E., Duron O., Kelly B.M. (1963). Improved method for the determination of blood glutathione. J. Lab. Clin. Med..

[56] Fikry E.M., Gad A.M., Eid A.H., Arab H.H. (2019). Caffeic acid and ellagic acid ameliorate adjuvant-induced arthritis in rats via targeting inflammatory signals, chitinase-3-like protein-1 and angiogenesis. Biomed. Pharmacother..

[57] Arab H.H., Ashour A.M., Gad A.M., Mahmoud A.M., Kabel A.M. (2021). Activation of AMPK/mTOR-driven autophagy and inhibition of NLRP3 inflammasome by saxagliptin ameliorate ethanol-induced gastric mucosal damage. Life Sci..

